# *Moringa oleifera* Flower Extract Suppresses the Activation of Inflammatory Mediators in Lipopolysaccharide-Stimulated RAW 264.7 Macrophages via NF-*κ*B Pathway

**DOI:** 10.1155/2015/720171

**Published:** 2015-11-02

**Authors:** Woan Sean Tan, Palanisamy Arulselvan, Govindarajan Karthivashan, Sharida Fakurazi

**Affiliations:** ^1^Laboratory of Vaccines and Immunotherapeutics, Institute of Bioscience, Universiti Putra Malaysia, 43400 Serdang, Selangor, Malaysia; ^2^Department of Human Anatomy, Faculty of Medicine and Health Sciences, Universiti Putra Malaysia, 43400 Serdang, Selangor, Malaysia

## Abstract

*Aim of Study*. *Moringa oleifera* Lam. (*M. oleifera*) possess highest concentration of antioxidant bioactive compounds and is anticipated to be used as an alternative medicine for inflammation. In the present study, we investigated the anti-inflammatory activity of 80% hydroethanolic extract of *M. oleifera* flower on proinflammatory mediators and cytokines produced in lipopolysaccharide- (LPS-) induced RAW 264.7 macrophages. *Materials and Methods*. Cell cytotoxicity was conducted by 3-(4,5-dimethylthiazol-2-yl)-2,5-diphenyltetrazolium bromide (MTT) assay. Nitric oxide (NO) production was quantified through Griess reaction while proinflammatory cytokines and other key inflammatory markers were assessed through enzyme-linked immunosorbent assay (ELISA) and immunoblotting. *Results*. Hydroethanolic extract of *M. oleifera* flower significantly suppressed the secretion and expression of NO, prostaglandin E_2_ (PGE_2_), interleukin- (IL-) 6, IL-1*β*, tumor necrosis factor-alpha (TNF-*α*), nuclear factor-kappa B (NF-*κ*B), inducible NO synthase (iNOS), and cyclooxygenase-2 (COX-2). However, it significantly increased the production of IL-10 and I*κ*B-*α* (inhibitor of *κ*B) in a concentration dependent manner (100 *μ*g/mL and 200 *μ*g/mL). *Conclusion*. These results suggest that 80% hydroethanolic extract of *M. oleifera* flower has anti-inflammatory action related to its inhibition of NO, PGE_2_, proinflammatory cytokines, and inflammatory mediator's production in LPS-stimulated macrophages through preventing degradation of I*κ*B-*α* in NF-*κ*B signaling pathway.

## 1. Introduction

The inflammatory process is consecutive and well-regulated mechanisms which respond to the stimulation and activation of the defense systems. The target cells such as macrophages have been stimulated by physical, chemical, microbial, and immunological reaction which produce inflammatory responses [1]. Inflammation is the central features of many chronic diseases which cause morbidity and mortality. The occurrence of chronic diseases has triggered prolonged inflammation that induced the expression of robust proinflammatory mediators and cytokines, which are harmful, which leads to the pathogenesis of inflammation associated chronic diseases [2].

*Moringa oleifera *Lam. (*M. oleifera*) family of Moringaceae is indigenous to India, Pakistan, Bangladesh, and Afghanistan, which is now widely distributed in many countries of the tropics and subtropics over the world [3].* M. oleifera* is a perennial angiosperm plant, and it is one among thirteen species belonging to the monogeneric family [4]. The bioactive compounds from various parts of the plant including leaves, roots, bark, gum, flowers, fruits, seeds, and seed oil have been attributed to high nutrition value and prophylactic and medicinal virtue [5]. Edible parts of* M. oleifera *have shown various pharmacological properties: mainly, antimicrobial, antihypercholesterolemic, antitumor, antidiabetic, and antioxidant properties [6, 7]. The medicinal importance of different parts of the plant including leaves, roots, seeds, and fruits has long been used as folkloric medicine to treat various ailments related to inflammation [8, 9]. Currently,* M. oleifera* have been interesting for many biomedical researchers due to the presence of bioactive compounds which are responsible for various biomedical applications. However, only few scientific findings have reported the biomedical application of* M. oleifera* flower extract; thus, we are interested in exploring its therapeutic potential as anti-inflammatory agents.

Lipopolysaccharide (LPS) is a principal component of the outer membrane of Gram-negative bacteria that can activate immunological responses in cells [10]. LPS activates the inflammatory mechanisms through three pathways which are mitogen-activated protein kinases (MAPKs), nuclear factor-kappa B (NF-*κ*B) signaling, and janus kinase/signal transducers and activators of transcription (JAK/STAT) pathways [11, 12]. NF-*κ*B signaling pathway is one of the highly expressed pathways among all other pathways, which enhanced various inflammatory genes expression (NF-*κ*B), inducible nitric oxide synthase (iNOS), cyclooxygenase-2 (COX-2), and production proinflammatory mediators (interleukin- (IL-) 6, IL-1*β*, and tumor necrosis factor-alpha (TNF-*α*)) [13–16]. Therefore, in the present study, we have investigated and reported the anti-inflammatory potential of 80% hydroethanolic extract of* M. oleifera* flower on producing various inflammatory mediators, NO, PGE_2_, IL-6, IL-1*β*, TNF-*α*, IL-10, NF-*κ*B, I*κ*B-*α*, COX-2, and iNOS, in LPS-stimulated murine macrophages through NF-*κ*B signaling pathway.

## 2. Materials and Methods

### 2.1. Chemical Reagents

Dulbecco's Modified Eagle's medium (DMEM), Fetal Bovine Serum (FBS), Penicillin/Streptomycin for cell culture, and Bovine Serum Albumin (BSA) and RIPA buffer were purchased from Nacalai (Kyoto, Japan). 3-(4,5-Dimethylthiazol-2-yl)-2,5-diphenyltetrazolium bromide (MTT), lipopolysaccharides from* Escherichia coli* 0111:B4 (LPS), and N-1-naphthylethylendiamide-dihydrochloride (NED) were obtained from Sigma-Aldrich Co. (St. Louis, MO, USA). Bicinchoninic acid (BCA) assay and sulphanilamide were obtained from Thermo Scientific (Waltham, MA, USA) and Friendemann Schmidt (CT Parkwood, WA, Australia), respectively. Primary antibodies specific to iNOS, COX-2, NF-*κ*B, I*κ*B-*α*, and *β*-actin were purchased from Santa Cruz Biotechnology (Santa Cruz, CA) and, in addition, anti-rabbit and/or anti-mouse secondary antibodies conjugated to horseradish peroxidase were obtained from Santa Cruz Biotechnology (Santa Cruz, CA).

### 2.2. Plant Material Collection and Extraction

The* M. oleifera* flowers were obtained from Garden No. 2 at Universiti Putra Malaysia and have been confirmed with the voucher specimen (SK 1561/08) that has been deposited in the IBS Herbarium unit. The flowers were washed, air-dried at room temperature for 12 h and oven-dried for two consecutive days at 45°C, grounded to powder form, and stored in vacuum bags*. M. oleifera* flower powder was macerated in hydroethanolic solvent (ethanol : distilled water, 80 : 20 [80%]) for 3 days under rotary shaker at room temperature. Further, the residue was filtered, solvent-evaporated, freeze-dried, weighed, and stored at 4°C until further investigation.

### 2.3. Chromatographic Analysis and Instrumentation

The analysis was carried out using a HPLC-UV system (Agilent 1100 series, USA) equipped with a binary pump, array detector (diode array detector [DAD]) (200 to 600 nm range; 5 nm bandwidth), and an autosampler. A LUNA C18 (4 × 250 mm, 5 *μ*m) Phenomenex column (Torrance, CA, USA) maintained at room temperature (25°C) was used in the chromatographic analysis. The separation was carried out in a gradient system with its mobile phase consisting of solvent A, distilled water, and solvent B, methanol : distilled water 70 : 30 (v/v). The gradient program profile was a combination of solvents A and B as follows: 0 to 10 min, 30% solvent B; 10 to 20 min, 40% solvent B; 20 to 35 min, 50% solvent B; 35 to 40 min, 60% solvent B; 40 to 45 min, 70% solvent B; and 45 to 50 min, 0% solvent B. The detection was made at 254 nm and the injection volume and flow rate were 20 *μ*L and 1.0 mL/min, respectively. The compounds in the hydroethanolic* M. oleifera* flower extracts were separated using a C18 column (4 × 250 mm, 5 *μ*m, Phenomenex) with a gradient mobile phase consisting of water (solvent A) and methanol with 1% acetonitrile (solvent B), each containing 0.1% formic acid and 5 mM ammonium format, using the gradient program of 40% solvent B to 50% solvent B over 11.00 min at a flow rate of 1.0 mL/min, and were identified with accurate mass detection using an AB Sciex 3200 QTrap LCMS/MS with a Perkin Elmer FX 15 UHPLC system (MA, USA). The sample injection volume was 20 *μ*L and the negative ion mass spectra were obtained with a LC QTrap MS/MS detector in full ion scan mode (100 to 1200* m/z* for full scan and 50–1200* m/z* for MS/MS scan) at a scan rate of 0.5 Hz. The system was supported with mass spectrometry software and a spectral library provided by ACD Labs (Toronto, ON, Canada). All chromatographic procedures were performed at ambient temperature, and the corresponding peaks from the QTrap LC MS/MS analysis of the compounds were identified by comparison with the literature/ACD Labs Mass Spectral Library.

### 2.4. Cell Culture

The murine macrophage cell line, RAW 264.7, was obtained from the American Type Culture Collection (ATCC, VA, USA) and maintained in DMEM supplemented with 10% heat-inactivated FBS and 1% penicillin/streptomycin at 37°C in a humidified incubator with 5% CO_2_. The cell's media were changed every 2-3 days and passaged in 70–90% confluent condition by trypsinization to maintain cells exponential growth stage.

### 2.5. 3-(4,5-Dimethylthiazol-2-yl)-2,5-diphenyltetrazolium Bromide (MTT) Colorimetric Assays

MTT assay was performed to determine the cytotoxicity and cell viability of 80% hydroethanolic* M. oleifera* flower extract on RAW 264.7 macrophages. The 100 *μ*L of RAW 264.7 macrophages was seeded in triplicate into 96-well plates (1 × 10^5^ cells/well) and incubated for 24 h. The macrophages were treated with various gradient concentration hydroethanolic flower extract with serial dilutions at 15.625, 31.25, 62.5, 125, 250, 500, and 1000 *μ*g/mL and then incubated for 24 h. Briefly, thereafter, 20 *μ*L of MTT solution (5 mg/mL) in phosphate-buffered solution (PBS) was added to each well and then followed by incubation for another 3 h. The medium was removed and the purple formazan crystals formed were dissolved by adding 100 *μ*L dimethyl sulfoxide (DMSO). The plate was swirled gently to mix well and kept in dark condition at room temperature for 30 min. The absorbance was determined by using ELx800 Absorbance Microplate Reader (BioTek Instruments Inc., VT, USA) at 570 nm wavelength. The results were expressed as a percentage of surviving cells over control cells.

### 2.6. Nitrite Quantification Assay

The NO was determined through the indication of nitrite level in the cell culture media. The macrophages were seeded in 6-well plates (1 × 10^6^ cells/well) with 2 mL of cell culture media and incubated for 24 h. This was followed by discarding the old culture media and replacing them with the new media to maintain the cells. Different concentrations of hydroethanolic* M. oleifera* flower extract (100 *μ*g/mL and 200 *μ*g/mL) and the positive control dexamethasone (0.5 *μ*g/mL) were pretreated with the RAW 264.7 macrophages. Induction of RAW 264.7 macrophages with LPS (1 *μ*g/mL) for all samples was conducted except in control for another 24 h. Then, 100 *μ*L of the collected supernatants was added with 100 *μ*L of Griess reagent (0.1% NED, 1% sulphanilamide, and 2.5% phosphoric acid) and incubated in room temperature for 10 min in dark condition. The absorbance was determined by using microplate reader at 540 nm wavelength. The NO concentration was determined by comparison to the standard curve.

### 2.7. Enzyme-Linked Immunosorbent Assay (ELISA) 

RAW 264.7 macrophages with or without hydroethanolic* M. oleifera* bioactive flower extract and dexamethasone (0.5 *μ*g/mL) in the presence of LPS (1 *μ*g/mL) were seeded in 6-well plates (1 × 10^6^ cells/well) for 24 h. RAW 264.7 macrophages untreated with LPS which act as control were included for comparison. The concentrations of PGE_2_ and cytokine mediators such as IL-6, IL-1*β*, TNF-*α*, and IL-10 were assayed in cultured media of macrophages using mouse ELISA kits (R&D Systems Inc., MN, USA), according to the manufacturer's instructions.

### 2.8. Immunofluorescence Staining

Macrophages (RAW 264.7 cells) were cultured in glass coverslips in 6-well plate (1 × 10^6^ cells/well) and inflammation induced by LPS with presence or absence of flower extract for 24 h and then fixed with methanol/acetone fixation. After that, fixed cells were permeabilized with 0.2% 10x Triton in PBS for 2 min at room temperature (RT). The macrophages in coverslips were then rinsed with PBS and incubated with (1% BSA in PBS) blocking buffer for 30 min at RT. The cells then incubated with NF-*κ*B primary antibody (1 : 250) and anti-rabbit secondary antibodies conjugated to fluorophores (1 : 1000) in blocking buffer for 1 h, respectively. Nuclear macrophages were stained with Hoechst (1 : 5000) from Thermo Scientific (Waltham, MA, USA) in PBS for 15 min. The macrophages were ready to view and photographs were taken through fluorescent microscope at 200x magnification (Olympus, Tokyo, Japan).

### 2.9. Immunoblot Analysis

Protein extracts were harvested and prepared by using RIPA buffer for Western blot analyses from treated macrophages. The concentration of protein was determined by using the BCA. Equal amounts of cellular proteins were loaded on 10% sodium dodecyl sulfate-polyacrylamide gel electrophoresis (SDS-PAGE) under reducing conditions for separation. The separated protein was then transferred to polyvinylidene difluoride (PVDF; GE Healthcare) membranes for 1 h. The membrane underwent blocking step for minimum 1 h with blocking solution (5% of BSA in phosphate-buffered saline containing 1% Tween-20 (PBST)) at room temperature prior to incubation of specific primary antibodies such as NF-*κ*B, I*κ*B-*α*, iNOS, COX-2, and *β*-actin at 4°C overnight. The membrane was washed 5 times with PBST followed by incubation with respective anti-rabbit or anti-mouse secondary antibodies conjugated to horseradish peroxidase for 1 h and washed 5 times with PBST for 10 min each. The bands were visualized using chemiluminescence system (Chemi Doc, Bio Rad, USA). The bands were followed by analysis using Image J software (Bio Techniques, New York, USA).

### 2.10. Statistical Analysis

The results were summarized from three independent experiments and data expressed as the mean ± standard deviation (SD). The significant differences were examined using IBM with SPSS 20.0 software (SPSS Inc., Chicago, USA). One-way analysis of variance (ANOVA) and Turkey's post hoc test were used for pairwise comparisons. *p* value of 0.05 or less was considered as statistically significant.

## 3. Results

### 3.1. Phytochemical Analysis of* M. oleifera* Flower Extract

**Figure 1:**
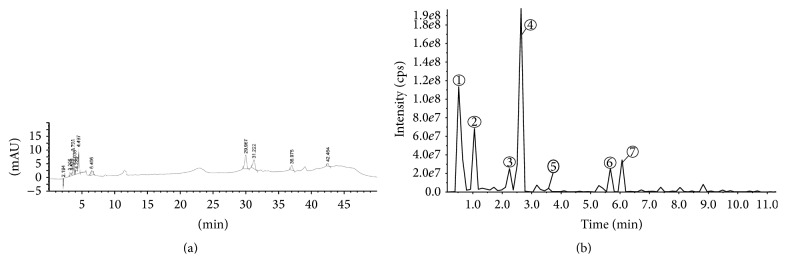
(a) HPLC-DAD (254 nm) fingerprints and (b) LC-MS/MS (254 nm) chromatogram of* M. oleifera* hydroethanolic flower extract.

To further interpret the observed effects of the* M. oleifera* flower extract, it is important to understand the molecular composition of the extract. In this regard, the HPLC fingerprint of 80% hydroethanolic* M. oleifera* flower extract (Figure 1(a)) was obtained to screen its peaks, followed by identification of compounds by LC-MS analysis (Figure 1(b)). Among the seven identified compounds, majority of the compounds were documented as phenolic compounds. Tentatively, these compounds have been identified and reported as quinic acid, 4-*p*-coumaroylquinic acid, quercetin-3-*O*-acetyl glucoside, kaempferol-3-*O*-acetyl hexoside, octadecenoic acid, heneicosanoic acid, and docosanoic acid and inclusive of other details such as *m*/*z* values and retention time, which were reported in (Table 1), based on the literature [7, 17–21]/ACD Labs Mass spectral Library.

### 3.2. Effect of* M. oleifera* on Cell Viability

**Figure 2:**
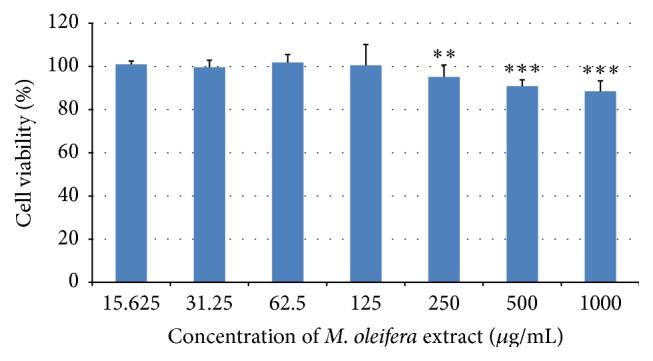
Effects of 80% hydroethanolic* M. oleifera* bioactive flower extract on the viability of RAW 264.7 macrophages. A density of 1 × 10^5^ cells/well of macrophages were seeded in 96-well plate and incubated with various concentrations of flower extract for 24 h. Cell viability was determined by MTT assay. The data are presented as mean ± SD of three independent experiments. ^*∗∗∗*^ *p* < 0.001, ^*∗∗*^ *p* < 0.01 versus culture media without flower extract which act as control.

MTT reduction assay was used to access the cytotoxicity effect of 80% hydroethanolic* M. oleifera* flower extract at concentration ranging from the lowest to highest (15.625–1000 *μ*g/mL) on RAW 264.7 macrophages. The cytotoxicity potential of flower extract on macrophages was presented in Figure 2. The results showed that increasing concentrations of hydroethanolic* M. oleifera* flower extract have caused reduction of cell viability. However, hydroethanolic* M. oleifera* flower extract did not exhibit any toxicity to macrophages at concentrations ranging from 15.625 to 125 *µ*g/mL. According to the cytotoxicity investigations, the concentrations at 100 *μ*g/mL and 200 *μ*g/mL were chosen for further anti-inflammatory experiments.

### 3.3. Effect of* M. oleifera* on NO Production

**Figure 3:**
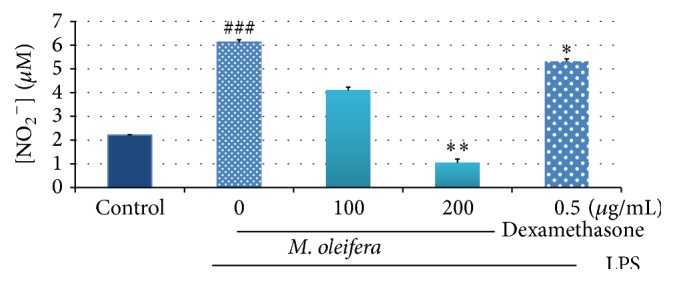
Effects of 80% hydroethanolic* M. oleifera* bioactive flower extract on NO production by LPS-induced RAW 264.7 macrophages. A density of 1 × 10^6^ cells/well of macrophages in the presence or absence of LPS were seeded in 6-well plate and treated with indicated concentrations of flower extract and dexamethasone for 24 h. The supernatants were collected and investigated by Griess assay. The data are presented as mean ± SD of three independent experiments. Control; basal level of nitrite released without LPS induction. ^###^ *p* < 0.001: LPS-treated group versus control; ^*∗∗*^ *p* < 0.01 and ^*∗*^ *p* < 0.05: treated group significantly different from LPS-treated group.

The effect of 80% hydroethanolic* M. oleifera* bioactive flower extract on NO production in LPS-induced RAW 264.7 macrophages was tested with NO assay. Griess reagent was used to determine nitrite (NO_2_^−^) released in the cell culture supernatant. Result from Figure 3 showed that the untreated control group released low level of nitrite (2.21 ± 0.016 *μ*M), while treated LPS group promoted nitrite production (6.120 ± 0.110 *μ*M) in inflammatory nature. The two different concentrations (at concentrations 100 *μ*g/mL and 200 *μ*g/mL) of 80% hydroethanolic flower extract gave good inhibitory effect on nitrite production. Dexamethasone, which was used as positive control, has also reduced the nitrite production (5.316 ± 0.106 *μ*M).* M. oleifera* extract treatment with 100 *μ*g/mL has decreased the nitrite secretion into 4.098 ± 0.133 *μ*M while 200 *μ*g/mL induced more attenuation effect on nitrite production (1.051 ± 0.149 *μ*M).

### 3.4. Effect of* M. oleifera* on PGE_2_ and Proinflammatory Cytokines Production

**Figure 4:**
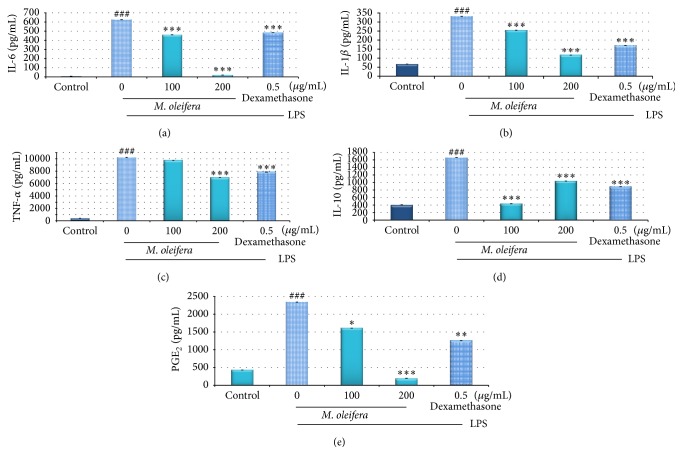
Effect of 80% hydroethanolic* M. oleifera* bioactive flower extracts on the production of cytokines IL-6, IL-1*β*, TNF-*α*, IL-10, and PGE_2_ by LPS-induced RAW 264.7 macrophages. A density of 1 × 10^6^ cells/well of macrophages induced by LPS were seeded in 6-well plate and treated with indicated concentrations of flower extract and dexamethasone for 24 h. The supernatants were collected and analysed by ELISA kits. The data are presented as mean ± SD of three independent experiments. ^###^ *p* < 0.001: LPS-treated group versus control; ^*∗∗∗*^ *p* < 0.001, ^*∗∗*^ *p* < 0.01, and ^*∗*^ *p* < 0.05: treated group significantly different from LPS-treated group. Control: basal level of cytokines released without LPS induction.

LPS-induced RAW 264.7 macrophages were used to determine the inhibitory action of 80% hydroethanolic* M. oleifera* flower extract on the production of PGE_2_ and proinflammatory enhancement of anti-inflammatory cytokines which was shown in Figures 4(a)–4(d): proinflammatory cytokines include IL-6, IL-1*β*, and TNF-*α*, while anti-inflammatory cytokine includes IL-10.  Figure 4(e) showed increased production of PGE_2_ in macrophages whereas these levels were suppressed while being treated with* M. oleifera* flower extract. LPS induction had trigged the production of all types of proinflammatory cytokines in macrophages.* M. oleifera* flower extract at concentration 200 *μ*g/mL treatment significantly reduced the production of IL-6 (19.083 ± 0.003 pg/*µ*L), IL-1*β* (116.889 ± 0.002 pg/*µ*L), and TNF-*α* (6840.5 ± 0.016 pg/*µ*L) but slightly increased production of IL-10 (1036 ± 0.002 pg/*µ*L) from 436 ± 0.0067 pg/*µ*L at concentration of 100 *μ*g/mL flower extract in the LPS-stimulated macrophages.

### 3.5. Effect of* M. oleifera* on NF-*κ*B p65 Expression

**Figure 5:**
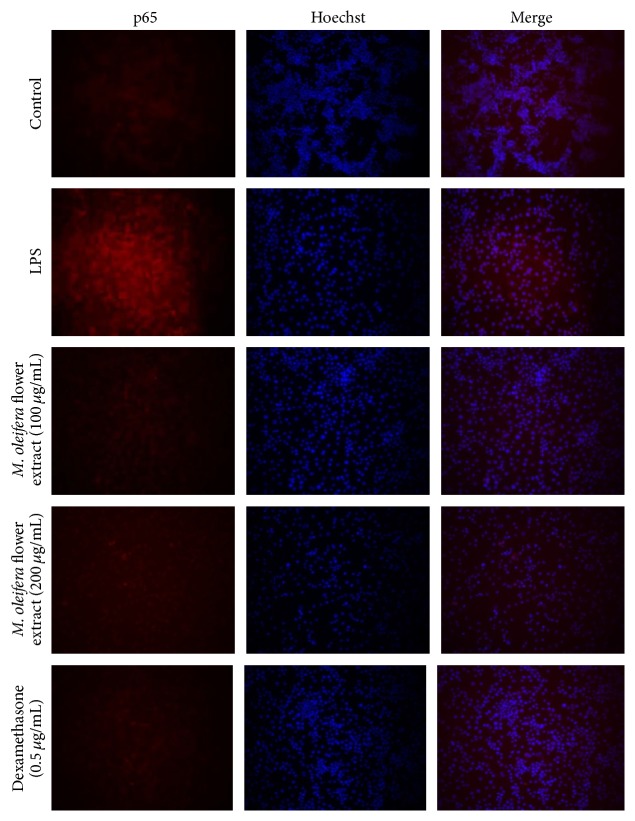
The effect of* M. oleifera* flower extract on NF-*κ*B p65 expression in LPS-stimulated RAW 264.7 macrophages. Macrophages were treated with extract (100 and 200 *µ*g/mL) and dexamethasone (0.5 *μ*g/mL) in the presence of LPS (1 *µ*g/mL) for 24 hours. Expression of NF-*κ*B p65 was observed by fluorescence microscope after immunofluorescence staining with anti-NF-*κ*B p65 antibody and fluorescein labeled anti-rabbit IgG (red). Nuclei of the cells were stained with Hoechst 33342 (blue) and images were captured (original magnification, ×200).

Immunofluorescence staining and fluorescence microscopy were used to examine the effect of* M. oleifera *flower extract on NF-*κ*B activation. As Figure 5 shows, the higher expression of NF-*κ*B activation was observed in LPS-stimulated macrophages; NF-*κ*B p65 were translocated from cytoplasm into nucleus. However, pretreatment with flower extract with concentrations of 100 and 200 *μ*g/mL suppressed/inhibited the LPS-induced NF-*κ*B p65 activation. These investigations were consistent with Western blot results indicating that* M. oleifera *flower extract effectively suppressed LPS-induced NF-*κ*B p65 expression in a concentration dependent manner.

### 3.6. Effect of* M. oleifera* on Expression of Inflammatory Mediators

**Figure 6:**
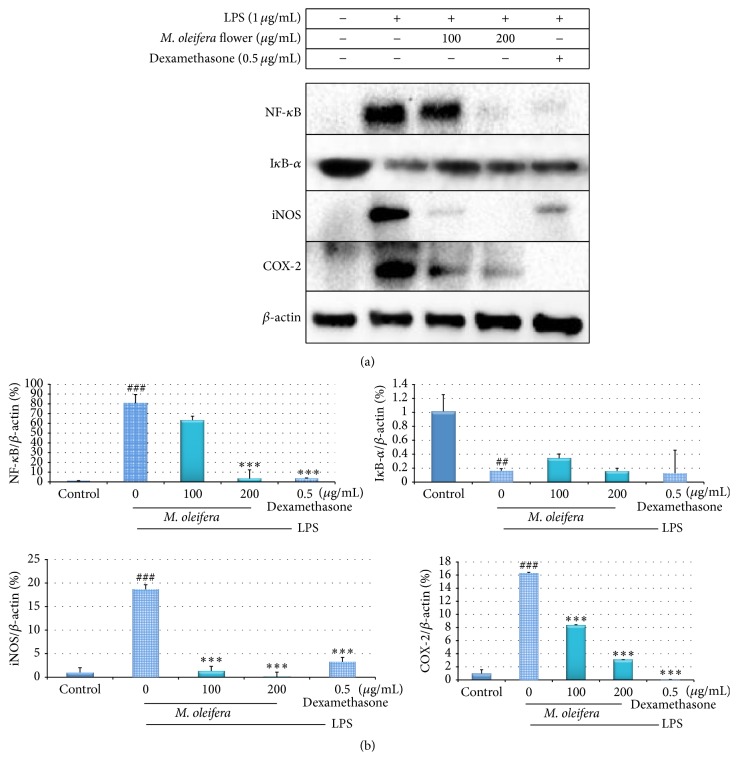
(a) Anti-inflammatory effect of 80% hydroethanolic* M. oleifera* bioactive flower extract on the expression of NF-*κ*B, I*κ*B-*α*, iNOS, and COX-2 in LPS-induced RAW 264.7 macrophages. A density of 1 × 10^6^ cells/well of macrophages in the presence or absence of LPS were seeded in 6-well plate and treated with indicated concentrations of flower extract for 24 h. The protein of cells was collected through RIPA buffer and analysed by Western blotting. *β*-actin acts as a loading control and also standard for target proteins in quantitative determination. (b) Densitometry analysis results of the effect of* M. oleifera *flower extract on proteins expression. ^###^ *p* < 0.001 and ^##^ *p* < 0.01 were LPS-treated group versus control; ^*∗∗∗*^ *p* < 0.001: treated group significantly different from LPS-treated group. Control: basal level of cytokines released without LPS induction. The data are presented as mean ± SD of three independent experiments.

Immunoblotting was conducted to evaluate the expression of inflammatory mediators which included NF-*κ*B, I*κ*B-*α*, iNOS, and COX-2 in LPS-stimulated RAW 264.7 macrophages treated with the 80% hydroethanolic* M. oleifera* flower extract at concentrations 100 and 200 *μ*g/mL. As illustrated in Figure 6, the NF-*κ*B, iNOS, and COX-2 target markers are significantly expressed in the LPS-treated group compared to the control untreated group. However, the treatment of* M. oleifera* flower extract concentration dependently downregulated the target molecule expressions in LPS-stimulated macrophages. On the other hand, I*κ*B-*α* expression is increased with the presence of flower extract.

## 4. Discussion

In recent years, utilization of plant-derived constituents in the field of pharmaceutical research arena has been increased abundantly, due to its wide array of medicinal properties and minimal or null toxicity compared with the synthetic drugs. Among traditional medicine,* M. oleifera* is well known for its impressive range of medicinal and nutritional value. Edible parts of this plant contain a high content of essential minerals, proteins, nutrients, and also various phenolic compounds stands for its medicinal properties. The leaves of this plant have been extensively investigated and certainly reported for its therapeutic potential and mechanism of action against various clinical complications, due to presence of rich bioactive candidates. Currently,* M. oleifera* flower has also been in the pipeline of investigation against hepatotoxicity, microbial infection, and other medical complications, which revealed positive reports [22–24]. However, only a few reports exist on the therapeutic potential of* M. oleifera* flower extract. Thus, in this study, we intended to evaluate the anti-inflammatory potential of* M. oleifera* flower extract and identify its liable active candidates through various chromatographic techniques.

Previously, our research team has reported that* M. oleifera* leaves are enriched with flavonoids such as kaempferol and quercetin [7] and also reported the presence of high flavonol contents in* M. oleifera* flowers grown at South Africa [25]. Accordingly, the results of this study also indicated that* M. oleifera* flower extract is enriched with major phenolic compounds such as quercetin and kaempferol. Hämäläinen et al. [26] and García-Mediavilla et al. [27] reported the anti-inflammatory potential of quercetin and kaempferol by inhibition of signal transducer and activator of transcription 1 (STAT-1) and NF-*κ*B pathway. These reports strongly suggested that the presence of quercetin and kaempferol in* M. oleifera *flower extract is supposedly responsible for its elevated anti-inflammatory activity. Despite other phenolic compounds such as quinic acid, 4-*p*-coumaroylquinic acid which has been previously reported* in M. oleifera* leaves is recently found to be present as of GC-MS/MS results on* M. oleifera* flower [28]. Accordingly, we identified the existence of quinic acid and 4-p-coumaroylquinic acid in* M. oleifera* flower extract, also evidently involved in its anti-inflammatory potential [29]. Apart from the phenolic compounds, few fatty acids/their derivatives have also been identified in* M. oleifera* flower extract. Fatty acids such as *α*-linolenic acid, oleic acid, octadecenoic acid, palmitic acid, heneicosanoic acid, capric acid, and behenic acid have already been reported to exist in* M. oleifera* leaves, root, and seed. However, to the best of our knowledge, we report here for the first time the presence of octadecenoic acid, heneicosanoic acid, and behenic acid in* M. oleifera* flower extract. Thus, from these reports, it can be concluded that the coexistence of major phenolic compounds and essential fatty acids is supposedly responsible for the enhanced anti-inflammatory potential of* M. oleifera* flower extract.

Raw 264.7 macrophages have been used as model to evaluate the effects of 80% hydroethanolic* M. oleifera *flower extract in anti-inflammatory activity due to phagocytic activities for immunological defence. Bacterial, viral, and fungal infection and tissue damage have caused the activation of proinflammatory signaling proteins especially toll-like receptors (TLRs). Macrophages produced various highly active proinflammatory mediators including the cytokines and chemokines like monocytes chemoattractant protein-1 (MCP1) and other inflammatory active molecules upon activation of TLRs [30]. Besides, inflammation involves induction of transcriptional mediators NF-*κ*B and activator protein 1 (AP-1), downstream from protein tyrosine kinases such as Syk and Src, serine/threonine kinases such as Akt, IKK, and TBK1, and mitogen-activated protein kinases [MAPKs: ERK (extracellular signal-related kinase), p38, and JNK (c-Jun N-terminal kinase)] [31].

LPS was bonded to toll-like receptor 4 (TLR-4) of macrophages and activated the downstream pathways which is signal transduction pathway kinases to induce inflammation via TLR-NF-*κ*B signaling pathways [10]. As shown in Figure 5, phosphorylation and degradation of I*κ*B-*α* in cytosol activated transcription factors and transferred NF-*κ*B into nucleus which caused increase in activity after stimulation with LPS. NF-*κ*B bonded to its response element and enhanced gene expression to produce proinflammatory cytokines and enzymes [32–34]. On activation, the level of cytokines (IL-6, IL-1*β*, TNF-*α*, and PGE_2_) (Figure 4) production in the culture supernatants was increased in response to LPS stimulation which showed the successful* in vitro* inflammation experimental model.

Mitochondrial dependent reduction of MTT colorimetric assay is one of* in vitro* assays to determine the potential cytotoxicity effect of flower extract. As the concentration of extract increased, the number of viable cells reduced. However, as shown in Figure 2,* M. oleifera *bioactive flower extract does not possess cytotoxicity effect on macrophages up to concentration 1000 *µ*g/mL since the cell viability is more than 80%. In this study, flower extract with concentrations 100 and 200 *µ*g/mL within the range of concentrations which give better cell viability percentages has been used for further* in vitro* anti-inflammatory investigations.

NO, a labile free radical gas, is an important mediator and regulator of inflammatory response and excessively generated during inflammation reaction [35]. The three types of isoforms of NO synthase (NOS) include neuronal NOS (nNOS), endothelial NOS (eNOS), and inducible NOS (iNOS). NO production in macrophages upon exposure to LPS is due to the oxidation of L-arginine into L-citrulline via the action iNOS in animal tissue [36, 37]. NO plays a role in vasodilatation, neurotransmission, and inhibition of platelet aggregation inflammation and induced cell apoptosis [38–40]. However, oversecretion of NO reacts with superoxide leading to tissues damage and contributes to pathological development of chronic inflammatory illnesses [41]. According to [29], licochalcone E (Lic E) suppressed the expression of iNOS and reduced the production of NO in dependent dose and showed it possesses potential anti-inflammatory effect. In present study, LPS-induced NO production (Figure 3) was significantly reduced by treatment with hydroethanolic* M. oleifera* flower extract via inhibiting iNOS expression (Figure 5) in a concentration dependent manner. Suppression of the iNOS and NO was observed after dexamethasone treatment in LPS-induced macrophages.

According to Makarov [42], increased production of proinflammatory cytokines such as TNF-*α*, IL-6, and IL-1*β* has resulted in adverse effect of inflammatory responses. Production of TNF-*α* mainly in macrophages via NF-*κ*B activation also stimulated the production of IL-1*β*, IL-6, and NO, thus acting as factor amplifying the inflammation and its associated complications [43]. According to [44], IL-6 is a B-cell differential factor which acts as multifunctional cytokine to regulate the immune and inflammatory response. Overproduction of IL-6 is often correlated with chronic diseases in inflammatory autoimmune diseases. However, IL-10 is an immunosuppressive, anti-inflammatory, and pleiotropic cytokine that modulates functions of immune cells. Treatments with hydroethanolic* M. oleifera* flower extract have suppressed the LPS-induced production of IL-6, IL-1*β*, and TNF-*α* but enhanced IL-10 by concentration dependently (Figures 4(a)–4(d)). Treatment with dexamethasone also revealed the inhibition on proinflammatory cytokines production but enhancement in IL-10 level in LPS-induced macrophages (^*∗∗∗*^ *p* < 0.001).

**Figure 7:**
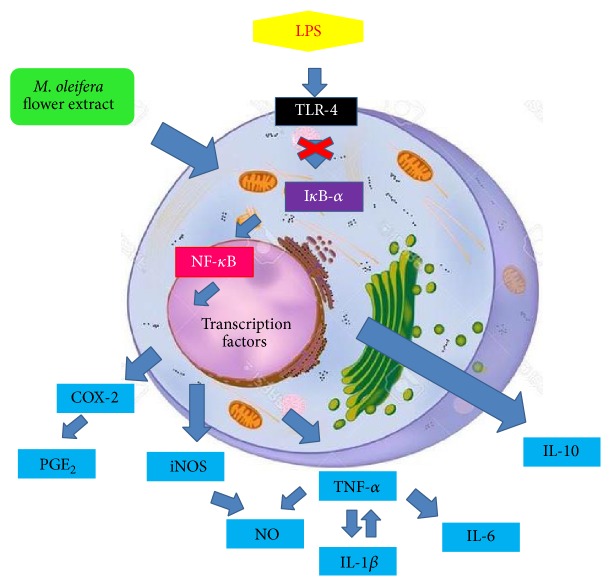
Mechanism blockade of NF-*κ*B activation in RAW 264.7 macrophages by 80% hydroethanolic* M. oleifera* flower extract.

NF-*κ*B is critical regulator mediator for iNOS, COX-2 transcription, and the production cytokines in LPS-induced macrophages. Inactive NF-*κ*B is located in cytoplasm as part of complex but activated NF-*κ*B upon LPS translocated to nucleus and bonded to its cognate DNA-binding sites to stimulate several intracellular signaling pathways [36]. This increases the expression of iNOS and COX-2 during inflammation [45]. Overexpressed iNOS in macrophages caused overproduced NO which induced inflammatory response. High expression of COX-2, an inducible enzyme which induced excessive production of PGE_2_, which act as proinflammatory mediators in inflammatory state [46]. The production of cytokines is regulated by NF-*κ*B expression through I*κ*B-*α* phosphorylation by I*κ*B kinase complex (IKK) [10, 47, 48]. Immunoblot results have (Figure 6) shown that LPS induces the degradation of I*κ*B-*α* expression by IKK complex, while* M. oleifera* flower extract and positive control treatment showed significantly enhanced expression of I*κ*B-*α*. Hydroethanolic* M. oleifera* flower extract and dexamethasone have exhibited anti-inflammatory properties in a concentration dependent fashion in suppressing LPS-induced production of proinflammatory mediators including IL-6, IL-1*β*, and TNF-*α*, as well as NF-*κ*B, iNOS, and COX-2 expression. However, they enhanced production of IL-10 and expression of I*κ*B-*α*. These results have proven that hydroethanolic* M. oleifera* flower extract exerted its activity on upstream signaling pathway.* M. oleifera* flower extract might inhibit NF-*κ*B activation activity by blocking the degradation of I*κ*B-*α* and retained NF-*κ*B in cytoplasm from further activation. Proinflammatory genes expressions from downstream targets of NF-*κ*B have been downregulated [8]. In this study, blockade of NF-*κ*B activation by inhibiting LPS-induced I*κ*B-*α* phosphorylation is an effective molecular target to prevent elevation of proinflammatory mediators as the mechanism shown in Figure 7.

## 5. Conclusion

In conclusion, we demonstrated that 80% hydroethanolic* M. oleifera* flower extract has significant effect on inhibiting the production of NO and downregulated the expression of inflammatory mediators (NF-*κ*B, iNOS, and COX-2) and proinflammatory cytokines (TNF-*α*, IL-1*β*, IL-6, and PGE_2_) whereas it increased expression of anti-inflammatory cytokines, IL-10 and I*κ*B-*α*, in LPS-stimulated macrophages. These findings suggest that 80% hydroethanolic* M. oleifera* flower extract can be a potent inhibitor of inflammation through NF-*κ*B signaling pathway. Further studies are needed to understand the precise molecular mechanisms regulating the anti-inflammatory activity in animal model and validate it as a modulator of macrophage activation.

## Figures and Tables

**Table 1:** Retention times, MS, and MS fragments of the major bioactive constituents present in hydroethanolic *M*. *oleifera* crude flower extract by HPLC–DAD–ESI–MS/MS.

Peak	Retention time (RT)	Molecular ion peak (M−H)^−^	MS^2^ fragment ions intensity	Tentative compounds identified
1	0.53	191	173, 127, 93 (100), 85	Quinic acid
2	1.05	337	191, 163, 119 (100)	4-*p*-Coumaroylquinic acid
3	2.24	506	300 (100), 271, 255, 179, 151	Quercetin-3-*O*-acetyl glucoside
4	2.63	490	284/286, 255 (100), 227	Kaempferol-3-*O*-acetyl hexoside
5	3.57	329	229, 211 (100), 171, 99	Octadecenoic acid
6	5.67	325	281, 253, 225, 183 (100)	Heneicosanoic acid
7	6.07	339	275, 239, 199, 183 (100)	Behenic (docosanoic) acid

## References

[1] Heo S.-J., Jang J., Ye B.-R. (2014). Chromene suppresses the activation of inflammatory mediators in lipopolysaccharide-stimulated RAW 264.7 cells. *Food and Chemical Toxicology*.

[2] Dilshara M. G., Jayasooriya R. G. P. T., Kang C.-H. (2013). Downregulation of pro-inflammatory mediators by a water extract of *Schisandra chinensis* (Turcz.) Baill fruit in lipopolysaccharide-stimulated RAW 264.7 macrophage cells. *Environmental Toxicology and Pharmacology*.

[3] Staples G. W., Herbst D. R. (2005). *A Tropical Garden Flora: Plants Cultivated in the Hawaiian Islands and Other Tropical Places*.

[4] Lambole V., Kumar U. (2012). Effect of Moringa oleifera Lam. on normal and dexamethasone suppressed wound healing. *Asian Pacific Journal of Tropical Biomedicine*.

[5] Mbikay M. (2012). Therapeutic potential of *Moringa oleifera* leaves in chronic hyperglycemia and dyslipidemia: a review. *Frontiers in Pharmacology*.

[6] Fakurazi S., Sharifudin S. A., Arulselvan P. (2012). Moringa oleifera Hydroethanolic extracts effectively alleviate acetaminophen-induced hepatotoxicity in experimental rats through their antioxidant nature. *Molecules*.

[7] Karthivashan G., Tangestani Fard M., Arulselvan P., Abas F., Fakurazi S. (2013). Identification of bioactive candidate compounds responsible for oxidative challenge from hydro-ethanolic extract of moringa oleifera leaves. *Journal of Food Science*.

[8] Muangnoi C., Chingsuwanrote P., Praengamthanachoti P., Svasti S., Tuntipopipat S. (2012). Moringa oleifera pod inhibits inflammatory mediator production by lipopolysaccharide-stimulated RAW 264.7 murine macrophage cell lines. *Inflammation*.

[9] Cheenpracha S., Park E.-J., Yoshida W. Y. (2010). Potential anti-inflammatory phenolic glycosides from the medicinal plant *Moringa oleifera* fruits. *Bioorganic and Medicinal Chemistry*.

[10] Guha M., Mackman N. (2001). LPS induction of gene expression in human monocytes. *Cellular Signalling*.

[11] Cruz M. T., Duarte C. B., Gonçalo M., Carvalho A. P., Lopes M. G. (1999). Involvement of JAK2 and MAPK on type II nitric oxide synthase expression in skin-derived dendritic cells. *The American Journal of Physiology—Cell Physiology*.

[12] Okugawa S., Ota Y., Kitazawa T. (2003). Janus kinase 2 is involved in lipopolysaccharide-induced activation of macrophages. *The American Journal of Physiology—Cell Physiology*.

[13] Ajizian S. J., English B. K., Meals E. A. (1999). Specific inhibitors of p38 and extracellular signal-regulated kinase mitogen-activated protein kinase pathways block inducible nitric oxide synthase and tumor necrosis factor accumulation in murine macrophages stimulated with lipopolysaccharide and interferon-*γ*. *Journal of Infectious Diseases*.

[14] An H.-J., Jeong H.-J., Um J.-Y., Kim H.-M., Hong S.-H. (2006). Glechoma hederacea inhibits inflammatory mediator release in IFN-*γ* and LPS-stimulated mouse peritoneal macrophages. *Journal of Ethnopharmacology*.

[15] Bhat N. R., Zhang P., Lee J. C., Hogan E. L. (1998). Extracellular signal-regulated kinase and p38 subgroups of mitogen-activated protein kinases regulate inducible nitric oxide synthase and tumor necrosis factor-alpha gene expression in endotoxin-stimulated primary glial cultures. *The Journal of Neuroscience*.

[16] Carter A. B., Monick M. M., Hunninghake G. W. (1999). Both Erk and p38 kinases are necessary for cytokine gene transcription. *American Journal of Respiratory Cell and Molecular Biology*.

[17] Aghofack-Nguemezi J., Fuchs C., Yeh S.-Y., Huang F.-C., Hoffmann T., Schwab W. (2011). An oxygenase inhibitor study in *Solanum lycopersicum* combined with metabolite profiling analysis revealed a potent peroxygenase inactivator. *Journal of Experimental Botany*.

[18] Marina K., Viktor G., Marina S. (2010). HPLC-DAD-ESI-MSn identification of phenolic compounds in cultivated strawberries from macedonia. *Macedonian Journal of Chemistry and Chemical Engineering*.

[19] Karthivashan G., Arulselvan P., Alimon A. R., Ismail I. S., Fakurazi S. (2015). Competing role of bioactive constituents in *Moringa oleifera* extract and conventional nutrition feed on the performance of cobb 500 broilers. *BioMed Research International*.

[20] Saldanha L. L., Vilegas W., Dokkedal A. L. (2013). Characterization of flavonoids and phenolic acids in *Myrcia bella* cambess. Using FIA-ESI-IT-MS^n^ and HPLC-PAD-ESI-IT-MS combined with NMR. *Molecules*.

[21] Singh P., Singh S. M., D'Souza L. M., Wahidullah S. (2012). Phytochemical profiles and antioxidant potential of four arctic vascular plants from Svalbard. *Polar Biology*.

[22] Sharifudin S. A., Fakurazi S., Hidayat M. T., Hairuszah I., Aris Mohd Moklas M., Arulselvan P. (2013). Therapeutic potential of *Moringa oleifera* extracts against acetaminophen-induced hepatotoxicity in rats. *Pharmaceutical Biology*.

[23] Pontual E. V., Napoleão T. H., de Assis C. R. D. (2012). Effect of *Moringa oleifera* flower extract on larval trypsin and acethylcholinesterase activities in *Aedes aegypti*. *Archives of Insect Biochemistry and Physiology*.

[24] Pontual E. V., De Lima Santos N. D., De Moura M. C. (2014). Trypsin inhibitor from *Moringa oleifera* flowers interferes with survival and development of *Aedes aegypti* larvae and kills bacteria inhabitant of larvae midgut. *Parasitology Research*.

[25] Pakade V., Cukrowska E., Chimuka L. (2013). Metal and flavonol contents of *Moringa oleifera* grown in South Africa. *South African Journal of Science*.

[26] Hämäläinen M., Nieminen R., Vuorela P., Heinonen M., Moilanen E. (2007). Anti-inflammatory effects of flavonoids: Genistein, kaempferol, quercetin, and daidzein inhibit STAT-1 and NF-*κ*B activations, whereas flavone, isorhamnetin, naringenin, and pelargonidin inhibit only NF-*κ*B activation along with their inhibitory effect on iNOS expression and NO production in activated macrophages. *Mediators of Inflammation*.

[27] García-Mediavilla V., Crespo I., Collado P. S. (2007). The anti-inflammatory flavones quercetin and kaempferol cause inhibition of inducible nitric oxide synthase, cyclooxygenase-2 and reactive C-protein, and down-regulation of the nuclear factor kappaB pathway in Chang Liver cells. *European Journal of Pharmacology*.

[28] Inbathamizh L., Padmini E. (2012). Gas chromatography-mass spectrometric analyses of methanol extract of *Moringa oleifera* flowers. *International Journal of Chemical and Analytical Science*.

[29] Lee S. Y., Moon E., Kim S. Y., Lee K. R. (2013). Quinic acid derivatives from *Pimpinella brachycarpa* exert anti-neuroinflammatory activity in lipopolysaccharide-induced microglia. *Bioorganic and Medicinal Chemistry Letters*.

[30] Toltl L. J., Swystun L. L., Pepler L., Liaw P. C. (2008). Protective effects of activated protein C in sepsis. *Thrombosis and Haemostasis*.

[31] Byeon S. E., Yi Y.-S., Oh J., Yoo B. C., Hong S., Cho J. Y. (2012). The role of Src kinase in macrophage-mediated inflammatory responses. *Mediators of Inflammation*.

[32] Aggarwal B. B., Natarajan K. (1996). Tumor necrosis factors: developments during the last decade. *European Cytokine Network*.

[33] Ahn K. S., Aggarwal B. B. (2005). Transcription factor NF-*κ*B: A sensor for smoke and stress signals. *Annals of the New York Academy of Sciences*.

[34] Medzhitov R., Kagan J. C. (2006). Phosphoinositide-mediated adaptor recruitment controls toll-like receptor signaling. *Cell*.

[35] MacMicking J., Xie Q.-W., Nathan C. (1997). Nitric oxide and macrophage function. *Annual Review of Immunology*.

[36] Choi E.-Y., Kim H.-J., Han J.-S. (2015). Anti-inflammatory effects of calcium citrate in RAW 264.7cells via suppression of NF-*κ*B activation. *Environmental Toxicology and Pharmacology*.

[37] Vane J. R., Mitchell J. A., Appleton I. (1994). Inducible isoforms of cyclooxygenase and nitric-oxide synthase in inflammation. *Proceedings of the National Academy of Sciences of the United States of America*.

[38] Coleman J. W. (2001). Nitric oxide in immunity and inflammation. *International Immunopharmacology*.

[39] Moncada S., Palmer R. M. J., Higgs E. A. (1991). Nitric oxide: physiology, pathophysiology, and pharmacology. *Pharmacological Reviews*.

[40] Kolb J. P., Paul-Eugene N., Damais C., Yamaoka K., Drapier J. C., Dugas B. (1994). Interleukin-4 stimulates cGMP production by IFN-*γ*-activated human monocytes. Involvement of the nitric oxide synthase pathway. *The Journal of Biological Chemistry*.

[41] Yang G.-Y., Taboada S., Liao J. (2009). Inflammatory bowel disease: a model of chronic inflammation-induced cancer. *Methods in Molecular Biology*.

[42] Makarov S. S. (2000). NF-*κ*B as a therapeutic target in chronic inflammation: recent advances. *Molecular Medicine Today*.

[43] Janssen-Heininger Y. M. W., Macara I., Mossman B. T. (1999). Cooperativity between oxidants and tumor necrosis factor in the activation of nuclear factor (NF)-*κ*B: requirement of Ras/mitogen-activated protein kinases in the activation of NF-*κ*B by oxidants. *American Journal of Respiratory Cell and Molecular Biology*.

[44] Yoon S.-B., Lee Y.-J., Park S. K. (2009). Anti-inflammatory effects of *Scutellaria baicalensis* water extract on LPS-activated RAW 264.7 macrophages. *Journal of Ethnopharmacology*.

[45] Lappas M., Permezel M., Georgiou H. M., Rice G. E. (2002). Nuclear factor Kappa B regulation of proinflammatory cytokines in human gestational tissues in vitro. *Biology of Reproduction*.

[46] Adelizzi R. A. (1999). COX-1 and COX-2 in health and disease. *Journal of the American Osteopathic Association*.

[47] Boyer L., Travaglione S., Falzano L. (2004). Rac GTPase instructs nuclear factor-*κ*B activation by conveying the SCF complex and IkB*α* to the ruffling membranes. *Molecular Biology of the Cell*.

[48] Chandel N. S., Trzyna W. C., McClintock D. S., Schumacker P. T. (2000). Role of oxidants in NF-*κ*B activation and TNF-*α* gene transcription induced by hypoxia and endotoxin. *Journal of Immunology*.

